# Disability Pension at the Time of Coronary Revascularisation Is Associated with Higher Five-Year Mortality; A Swedish Nationwide, Register-Based Prospective Cohort Study

**DOI:** 10.1371/journal.pone.0135277

**Published:** 2015-08-11

**Authors:** Katharina Zetterström, Margaretha Voss, Kristina Alexanderson, Torbjörn Ivert, Kenneth Pehrsson, Niklas Hammar, Marjan Vaez

**Affiliations:** 1 Department of Clinical Neuroscience, Division of Insurance Medicine, Karolinska Institutet, Stockholm, Sweden; 2 Department of Analysis and Forecasts, Swedish Social Insurance Agency, Stockholm, Sweden; 3 Department of Cardiothoracic Surgery and Anesthesiology, Karolinska University Hospital and Department of Molecular Medicine and Surgery, Karolinska Institutet, Stockholm, Sweden; 4 Department of Medicine, Karolinska Institutet, Stockholm, Sweden; 5 AstraZeneca R&D, Mölndal, Sweden; 6 Institute of Environmental Medicine, Karolinska Institutet, Stockholm, Sweden; 7 Centre for Occupational and Environmental Medicine, Stockholm County Council, Stockholm, Sweden; Azienda Ospedaliero-Universitaria Careggi, ITALY

## Abstract

**Background:**

Although coronary revascularisation by coronary artery bypass grafting (CABG) and percutaneous coronary intervention (PCI) are common procedures, little is known regarding disability pension (DP) at the time of coronary revascularisation and its association with mortality. The aim was to investigate the five-year mortality following a first coronary revascularisation among women and men on DP, compared with those not on DP at the time of intervention, accounting for socio-demographic and medical factors.

**Material and Methods:**

A nationwide prospective population-based cohort study was conducted, using national registers including 70,040 patients (80% men), aged 30–64 years, with a first CABG (n = 24,987; 36%) or PCI (n = 45,053; 64%) during 1994–2006 in Sweden, who were alive 30 days after the intervention. The main outcome was all-cause and cause-specific mortality within five years or through 31 December 2006, following CABG and PCI, and the exposure was DP at the time of a first coronary revascularisation. Information on DP, patient characteristics, date and cause of death was obtained from nationwide registers. Hazard ratios (HR) with 95% confidence intervals (CI) for the outcome were estimated, using Cox proportional hazard regression analyses. All analyses were stratified by type of intervention and gender.

**Findings:**

Four percent died following coronary revascularisation. Cardiovascular disease was the most common cause of death (54%), followed by neoplasms (25%). Regardless of type of intervention, gender and after multivariable adjustments, patients on DP had a higher HR for five-year mortality compared with those not on DP at time of revascularisation (CABG: women HR 2.14; 95% CI 1.59–2.89, men HR 2.09; 1.84–2.38, PCI: women HR 2.25; 1.78–2.83, men HR 1.95; 1.72–2.21). Young women on DP at the time of PCI had a substantially higher HR (HR 4.10; 95% CI: 2.25–7.48).

**Conclusion:**

Patients on DP at the time of first coronary revascularisation had a higher five-year risk of mortality compared with those not on DP.

## Introduction

During the last decades, disability pension (DP), in some countries called early retirement on medical grounds, or incapacity benefit, has become more common in many western countries [1–3]. In Sweden, cardiovascular diseases (CVD) constitute the third largest diagnostic group for DP [4–6]. Each year, about 10,000 working-aged women and men with ischemic heart disease undergo coronary revascularisation by coronary artery bypass graft surgery (CABG) or percutaneous coronary intervention (PCI). These established and well-investigated interventions [7–13] have resulted in relieved symptoms, improved physical capacity, and over the past decades, a continued decreased mortality, regardless of age and gender [11].

Previous studies have reported gender differences in mortality following coronary revascularisation; women have a higher short-term but a lower long-term mortality compared with men [14–17]. Moreover, 25% of all patients in Sweden with a first time coronary revascularisation in 1994–2003 had DP already at the time of the intervention. Of these patients, 62% had been on DP since many years before the intervention, and were, hence, already permanently excluded from the labour market. Women had a higher risk of being on DP at the time of intervention than men [18].

Previous studies of the general population have shown that individuals on DP have a higher risk of premature death than those not on DP; in spite of that most DP-diagnoses are non-lethal e.g., musculoskeletal and common mental disorders [19–25].

To the best of our knowledge, no studies have so far investigated risk of premature death among women and men on DP compared to those not on DP at their time of a first coronary revascularisation. The aim of this study was to investigate the five-year mortality following a first coronary revascularisation among women and men on DP compared with those not on DP at the time of intervention, accounting for socio-demographic and medical factors.

## Materials and Methods

### Ethics Statement

The study population was identified through nationwide registers collected and stored with informed consent of the patients. Additional information was collected by linkage of several public nationwide registers. In Sweden, ethical vetting is always required when using register data for the purpose of research. The ethical vetting is performed by regional ethical review boards and the risk appraisal associated with the Law on Public Disclosure and Secrecy is also done by data owners. The ethical review boards can, however, waive the requirement to consult the data subjects directly to obtain their informed consent, and will often do so if the research is supported by the ethical review board and the data has already been collected in some other context. According to these standards in Sweden this project has been evaluated and approved by the Regional Ethical Review Board of Stockholm, Sweden (2006/661-31).

### Study population

This population-based prospective cohort study based on register data, comprised all 70,040 (80% men) individuals in Sweden who during 1994–2006, when aged 30–64 years, had a first CABG (n = 24,987) or PCI (n = 45,053) and who were alive 30 days after the intervention. The patients were identified from the nationwide register for coronary revascularisation SWEDEHEART (Swedish Web-system for Enhancement and Development of Evidence-based care in Heart disease Evaluated According to Recommended Therapies) [26], including information on patient characteristics, date, and type of intervention for all performed coronary revascularisations in Sweden.

### Linkage to Nationwide Registers

The information from nationwide registers was obtained for all individuals through linkage, using the unique personal identification number given all residents in Sweden. Data on DP was obtained from the Swedish Social Insurance Agency (MiDAS data base); level of education, country of birth, and type of living area from Statistics Sweden (the LISA data base); date and cause of death through 31 December 2006 from the National Board of Health and Welfare (The Cause of Death Register), from which also diabetes mellitus and indication for intervention was obtained (The National Patient Register) if such data was not available in SWEDEHEART.

### Outcome

The outcome was all-cause and cause-specific mortality within five years or up through 31 December 2006, following a first coronary revascularisation by CABG or PCI.

Main cause of mortality was classified according to the International Classification of Disease 10 (ICD-10) into: CVD (I00-I99), neoplasms (C00-D48), endocrine, nutritional and metabolic disease (E00-E90), external causes (V01-V98), and others.

### Exposure

Being on DP (part-time: ≤50%, or full-time: >50%) at the time of a first coronary revascularisation. The reference category was those with no DP at the time of coronary revascularisation.

### Socio-demographic variables

Age at intervention: 30–49, 50–54, 55–59, and 60–64 years. Level of education: elementary school (≤9 years), high school (>9 and ≤12 years), college/university (>12 years). Country of birth: Sweden versus other countries. Type of living area: large cities, medium sized cities, and small communities.

### Medical variables

Year of intervention: 1994–1997, 1998–2001, and 2002–2006. Indication for intervention was categorized according to ICD-10 into: acute coronary syndrome (ACS) classified according to electrocardiographic findings as non-ST-segment elevation or ST-segment elevation myocardial infarction, stable angina, and other/missing. Diabetes mellitus at the time of intervention: yes, no, and missing data. In-patient care within the five years up to one day before date of intervention: yes versus no. Re-intervention: having or not having at least one re-intervention within the five year follow-up.

### Statistical analyses

Patient characteristics and cause-specific mortality were presented in frequencies and proportions. Further, we performed Cox proportional hazard analyses to calculate crude and adjusted hazard ratios (HR) with 95% confidence intervals (CI) for mortality within five years following a first coronary revascularisation among patients on DP or not on DP (reference group) at the time of intervention. The proportional hazard assumption was checked graphically and no indication of violation of this assumption was found. All individuals contributed with person time from the date of the first intervention (1994 through 31 November 2006) until the date of emigration or death, for five years after the first intervention or, (for those with an intervention later than 2001), until the end of 2006. Adjusted HR were calculated as: model I: age, and model II: age, educational level, country of birth, type of living area, year of intervention, indication for intervention, diabetes mellitus, in-patient care in the five years before intervention, and re-intervention (one or more) within five years following intervention. All analyses were stratified by type of intervention and gender; and the HR for mortality among those on DP, and not on DP at intervention were estimated within each subpopulation.

### Social insurance in Sweden

All individuals in Sweden aged 19–64 years with long-term or permanent work incapacity due to disease or injury can be granted DP. The common age for old-age pension is 65, but it can be obtained earlier. For individuals with no, or a low previous income, the DP benefits amount to a minimum level. For those with a previous income, the benefits amount to at least 64% of lost income, up to a certain level.

## Results

The study population (n = 70,040) consisted of CABG-patients (36%) and PCI-patients (64%) (Tables 1 and 2). A majority of the patients were 55 years or older (65%) at the time of intervention, had at most a high school education (73%) and were born in Sweden (82%). More patients lived in medium sized cities (37%) than in large cities and small communities and had their first coronary revascularisation within year 2002–2006 (46%). The majority had ACS as a main indication for intervention (60%). Eighteen percent (21% of all patients with diabetes data), had diabetes mellitus at time of their first intervention and 72% (74% among women, 72% among men) had had in-patient care in the five years before intervention. Most had been on DP for at least 4 years before the intervention (63%), and 17% (18% among women, 17% among men) had had at least one re-intervention within five years after the first intervention. Twenty-five percent (38% among women, 21% among men) had DP at the time of their first coronary revascularisation.

**Table 1:** Patient characteristics, and the five-year (or up through 2006) all-cause mortality, number (n) and percentages (%), of all patients in Sweden aged 30–64 years, on disability pension (DP) and not on DP at the time of first coronary artery bypass graft surgery (CABG) during the year 1994–2006, and alive 30 days after the intervention.

	CABG N = 24,987							
	Women n = 4046 (16.2%)				Men n = 20,941 (83.8%)			
	DP n	Death %^1^	No DP n	Death %^1^	DP n	Death %^1^	No DP n	Death %^1^
**All**	1691	8.2	2355	3.1	5032	9.7	15,909	3.5
**Age at intervention (years)**								
30–49	123	12.2	387	3.4	269	8.6	2281	2.1
50–54	210	8.6	436	2.3	589	9.0	3335	2.7
55–59	467	6.9	677	3.1	1383	9.0	5087	3.7
60–64	891	8.2	855	3.4	2791	10.2	6206	3.7
**Level of education**								
Elementary	904	8.7	863	3.7	2651	10.4	6049	3.9
High school	645	7.9	1075	3.1	1881	9.1	6625	3.4
College/University	132	6.1	384	1.8	460	7.4	3123	3.0
Missing data	10	0	33	3.0	40	10.0	112	6.3
**Country of birth**								
Sweden	1290	7.6	1952	2.5	3799	8.7	13,325	3.2
Other	401	10.0	403	6.0	1233	12.5	2584	5.1
**Type of living area (cities and communities)**								
Large	430	8.1	731	3.1	1433	9.7	5161	3.6
Medium	602	7.6	854	3.0	1758	9.8	5601	3.8
Small	659	8.6	770	3.1	1841	9.5	5147	3.1
**Year of intervention**								
1994–1997	653	9.0	890	4.2	2101	10.7	5802	4.2
1998–2001	516	9.9	782	3.5	1521	12.0	5174	3.8
2002–2006	522	5.4	683	1.3	1410	5.7	4933	2.4
**Indication for intervention**								
Acute Coronary Syndrome^2^	993	8.1	1313	42	2899	297	8572	299
Stable angina	611	8.2	920	2.9	1851	8.6	6461	3.2
Other/missing	89	9.0	122	3.3	282	10.6	876	6.3
**Diabetes mellitus**								
Yes	656	13.0	582	6.2	1433	14.2	2779	5.5
No	838	4.9	1470	1.8	2845	7.6	10,864	2.9
Missing data	197	6.1	303	3.6	754	8.9	2266	3.8
**In-patient care (in the five years before intervention)**								
Yes	1612	8.5	2119	3.4	4736	9.9	14,318	3.7
No	79	1.3	236	0.8	296	5.4	1591	2.3
**Years on DP before intervention**								
≤3	511	5.9	-		2059	8.8	-	
4–10	777	8.5	-		2238	10.0	-	
11–35	403	10.4	-		735	11.0	-	
**Re-intervention (at least one within five years)**								
Yes	115	7.0	136	2.2	194	7.7	738	2.6
No	1576	8.2	2219	3.2	4838	9.7	15,171	3.6

^1^ Percentage of all in each sub-group.

^2^ Non-ST-elevated or ST-elevated myocardial infarction.

**Table 2:** Patient characteristics, and the five-year (or up through 2006) all-cause mortality, number (n) and percentages (%), aged 30–64 years, on disability pension (DP) or not on DP at the time of first percutaneous coronary intervention (PCI) during the year 1994–2006 and alive 30 days after the intervention.

	PCI N = 45,053							
	Women n = 9979 (22.1%)				Men n = 35,074 (77.9%)			
	DP n	Death %^1^	No DP n	Death %^1^	DP n	Death %^1^	No DP n	Death %^1^
**All**	3703	5.8	6276	2.1	6714	7.0	28,360	2.6
**Age at intervention (years)**								
30–49	370	7.8	1298	1.7	650	5.5	6128	1.7
50–54	540	4.8	1367	1.0	976	4.8	6336	2.4
55–59	1062	4.4	1781	2.4	2002	7.4	8539	3.0
60–64	1731	6.4	1830	2.8	3086	7.7	7357	3.0
**Level of education**								
Elementary	1697	6.5	2023	2.8	3192	7.7	9469	3.0
High school	1630	5.0	2972	1.6	2778	6.6	12,414	2.6
College/University	350	4.9	1200	2.2	674	5.2	6328	2.0
Missing data	26	11.5	81	1.2	70	11.4	149	2.7
**Country of birth**								
Sweden	2871	4.6	5302	1.7	4938	6.6	23600	2.2
Other	832	9.7	974	4.3	1776	8.2	4760	4.4
**Type of living area (cities and communities)**								
Large	1028	6.3	1963	2.3	2039	6.8	8835	2.6
Medium	1465	5.7	2373	2.0	2498	7.5	10,835	2.8
Small	1210	5.4	1940	2.1	2177	6.6	8690	2.3
**Year of intervention**								
1994–1997	612	8.3	1131	2.1	1293	9.5	4793	3.7
1998–2001	994	7.1	1901	3.3	1727	9.2	7737	3.7
2002–2006	2097	4.3	3244	1.3	3694	5.1	15,830	1.7
**Indication for intervention**								
Acute Coronary Syndrome^2^	2276	0.8	3977	2.2	4135	6.1	14,999	2.8
Stable angina	1299	6.3	2140	1.9	2345	7.8	9479	3.0
Other/missing	128	10.2	159	1.9	234	15.0	882	4.1
**Diabetes mellitus**								
Yes	993	9.2	934	3.7	1537	11.3	3729	4.2
No	2360	4.4	4617	1.7	4449	5.3	21,604	2.1
Missing data	350	4.9	725	2.2	728	8.0	3027	4.1
**In-patient care (in the five years before intervention)**								
Yes	2788	6.8	3871	2.2	5009	8.5	16,201	3.4
No	915	2.6	2405	1.8	1705	2.6	12,159	1.5
**Years on DP before intervention**								
≤3	1168	3.9	-	-	2704	6.5	-	-
4–10	1609	5.7	-	-	2858	7.3	-	-
11–35	926	8.2	-	-	1152	7.3	-	-
**Re-intervention (at least one within five years)**								
Yes	878	5.8	1445	2.0	1774	7.4	6719	2.6
No	2825	5.7	4831	2.1	4940	6.9	21,641	2.6

^1^ Percentage of all in each sub-group.

^2^ Non-ST-elevated or ST-elevated myocardial infarction.

In the entire cohort regardless of type of intervention and gender, 4% (n = 2806) died within the follow-up. CVD was the most common cause of death (54%) followed by neoplasms (25%) and endocrine, nutritional and metabolic diagnoses (7%) (Table 3).

**Table 3:** Cause-specific mortality (n = 2806; 4.0%) within five years or up through 2006, after a first revascularisation by coronary artery bypass graft surgery (CABG) or percutaneous coronary intervention (PCI) within year 1994–2006, in all patients, aged 30–64 years, with disability pension (DP), n = 1307, and with no DP, n = 1499, at the time of intervention, and alive 30 days after intervention.

Cause-specific mortality									
	All	CABG N = 1257				PCI N = 1549			
		Women n = 211		Men n = 1046		Women n = 343		Men n = 1206	
	DP/No DP	DP	No DP	DP	No DP	DP	No DP	DP	No DP
	n (%)	n (%)	n (%)	n (%)	n (%)	n (%)	n (%)	n (%)	n (%)
**Cardiovascular disease (I00-I99)**	1509 (53.8)	62 (44.9)	33 (45.2)	282 (58.0)	318 (56.8)	107 (50.2)	58 (44.6)	273 (58.1)	376 (51.1)
**Neoplasms (C00-D48)**	710 (25.3)	29 (21.0)	22 (30.1)	99 (20.4)	136 (24.3)	45 (21.1)	57 (43.8)	93 (19.8)	229 (31.1)
**Endocrine, nutritional and metabolic disease (E00-E90)**	191 (6.8)	30 (21.7)	11 (15.1)	36 (7.4)	35 (6.3)	21 (9.9)	3 (2.3)	31 (6.6)	24 (3.3)
**External causes (V01-Y98)**	125 (4.5)	2 (1.4)	2 (2.7)	13 (2.7)	27 (4.8)	7 (3.3)	6 (4.6)	19 (4.0)	49 (6.7)
**Others**	271 (9.6)	15 (11.0)	5 (6.9)	56 (11.5)	44 (7.8)	33 (15.5)	6 (4.7)	54 (11.5)	58 (7.8)
**All**	2806	138	91	486	618	213	130	470	805

The HR for mortality was higher in patients on DP compared with those not on DP at the time of the first coronary revascularisation (CABG: women HR 2.73; 95% CI 2.06–3.63, men HR 2.64; 2.33–2.98, PCI: women HR 3.07; 2.47–3.82, men HR 2.16; 2.39–3.02), and remained higher regardless of type of intervention, gender, and adjustments for socio-demographic and medical factors (CABG: women HR 2.14; 95% CI 1.59–2.89, men HR 2.09; 95% CI 1.84–2.38, PCI: women HR 2.25; 95% CI 1.78–2.83, men HR 1.95; 95% CI 1.72–2.21) (Tables 4 and 5).

**Table 4:** Crude and adjusted hazard ratio (HR) with 95% confidence interval (CI) for all-cause mortality within five years following a first coronary artery bypass graft surgery (CABG), among women and men, with disability pension (DP) or no DP (reference group) at time of revascularisation, for all and within each subpopulation.

	Women N = 4493			Men N = 22,712		
	Crude	Model I	Model II	Crude	Model I	Model II
	HR (95% CI)	HR (95% CI)	HR (95% CI)	HR (95% CI)	HR (95% CI)	HR (95% CI)
**All**	2.73 (2.06–3.63)	2.77 (2.07–3.71)	2.14 (1.59–2.89)	2.64 (2.33–2.98)	2.36 (2.08–2.68)	2.09 (1.84–2.38)
**Age at intervention** (years) ^1^						
30–49	3.94 (1.88–8.28)		1.00 (0.39–2.54)	4.21 (2.54–6.95)		2.45 (1.43–4.20)
50–54	4.58 (2.11–9.94)		3.72 (1.62–8.57)	3.36 (2.39–4.71)		2.90 (2.04–4.13)
55–59	2.25 (1.30–3.89)		1.66 (0.94–2.93)	2.26 (1.80–2.83)		2.00 (1.58–2.54)
60–64	2.36 (1.54–3.63)		1.81 (1.16–2.81)	2.09 (1.76–2.49)		1.96 (1.64–2.34)
**Level of education**						
Elementary school	2.49 (1.66–3.74)	2.49 (1.65–3.76)	1.98 (1.30–3.02)	2.61 (2.20–3.10)	2.31 (1.94–2.76)	2.13 (1.78–2.55)
High school	2.78 (1.80–4.31)	2.76 (1.76–4.34)	2.18 (1.38–3.45)	2.62 (2.15–3.19)	2.41 (1.96–2.95)	2.13 (1.72–2.62)
College or university	3.52 (1.28–9.73)	4.64 (1.60–13.46)	1.00 (0.28–3.54)	2.37 (1.60–3.52)	2.15 (1.44–3.23)	1.90 (1.26–2.88)
**Country of birth**						
Sweden	3.17 (2.25–4.59)	3.25 (2.29–4.62)	2.50 (1.74–3.58)	2.59 (2.25–2.99)	2.32 (2.00–2.70)	2.11 (1.81–2.46)
Others	1.65 (0.96–2.74)	1.63 (0.96–2.75)	1.43 (0.83–2.46)	2.35 (1.86–2.97)	1.99 (1.57–2.54)	2.05 (1.61–2.62)
**Type of living area** (cities and communities)						
Large	2.64 (1.56–4.46)	2.83 (1.64–4.88)	2.36 (1.34–4.15)	2.60 (2.09–3.24)	2.37 (1.89–2.97)	2.10 (1.66–2.66)
Medium	2.58 (1.60–4.18)	2.55 (1.56–4.18)	2.02 (1.20–3.40)	2.49 (2.04–2.04)	2.17 (2.76–2.67)	1.87 (1.51–2.31)
Small	2.97 (1.84–4.78)	2.97 (1.82–4.83)	2.29 (1.40–3.76)	2.90 (2.34–3.59)	2.64 (2.11–3.31)	2.43 (1.93–3.06)
**Year of intervention**						
1994–1997	2.18 (1.44–3.28)	2.21 (1.45–3.38)	1.76 (1.14–2.72)	2.51 (2.09–3.01)	2.25 (1.86–2.71)	1.92 (1.58–2.34)
1998–2001	2.94 (1.94–4.69)	3.99 (1.84–4.84)	2.23 (1.36–3.66)	3.17 (2.59–3.88)	2.95 (2.39–3.63)	2.61 (2.10–3.24)
2002–2006	4.20 (1.98–8.90)	4.19 (1.85–9.02)	3.82 (1.70–8.57)	2.57 (1.94–3.42)	2.37 (1.78–3.15)	1.79 (1.33–2.40)
**Indication for intervention**						
Acute Coronary Syndrome	2.58 (1.78–3.75)	2.45 (1.68–3.59)	1.90 (1.28–2.83)	2.83 (2.41–3.32)	2.51 (2.12–2.96)	2.20 (1.85–2.61)
Stable angina pectoris	3.03 (1.90–4.84)	3.35 (2.06–5.45)	2.51 (1.52–4.12)	2.62 (2.13–3.23)	2.32 (1.87–2.88)	2.04 (1.63–2.54)
Other/missing	2.57 (0.77–8.56)	2.49 (0.70–8.88)	1.00 (0.26–3.86)	1.68 (1.08–2.63)	1.66 (1.05–2.61)	1.00 (0.59–1.69)
**Diabetes Mellitus**						
Yes	2.22 (1.51–3.28)	2.25 (1.51–3.35)	2.17 (1.45–3.25)	2.38 (1.93–2.93)	2.20 (1.77–2.74)	2.26 (1.81–2.81)
No	2.75 (1.69–4.50)	2.59 (1.56–4.29)	2.34 (1.39–3.93)	2.50 (2.11–2.98)	2.15 (1.80–2.57)	2.03 (1.69–2.44)
Missing data	1.82 (0.80–4.13)	1.80 (0.76–4.28)	1.09 (0.45–2.63)	2.27 (1.65–3.13)	2.10 (1.50–2.93)	1.00 (0.68–1.47)
**In-patient care** (in the five years before intervention)						
Yes	2.65 (1.99–3.53)	2.70 (2.16–3.78)	2.17 (1.61–2.94)	2.62 (2.31–2.97)	2.35 (2.06–2.67)	2.10 (1.84–2.39)
No	1.65 (0.15–18.21)	1.17 (0.11–12.87)	0.37 (0.01–9.44)	2.35 (1.31–4.23)	2.15 (1.17–3.96)	1.88 (1.01–3.49)
**Re-intervention** (at least one within five years)						
Yes	2.96 (0.79–11.18)	2.34 (0.61–8.93)	2.05 (0.50–8.44)	3.27 (1.66–6.44)	2.65 (1.30–5.40)	2.40 (1.12–5.17)
No	2.74 (2.06–3.67)	2.82 (2.09–3.80)	2.16 (1.60–2.94)	2.61 (2.30–2.95)	2.35 (2.07–2.67)	2.09 (1.83–2.38)

Model I = Adjusted for age; model II = Adjusted for: age, level of education, country of birth, type of living area, year of intervention, indication for intervention, diabetes mellitus, in-patient care within 5 years up to one day before intervention, and re-intervention.

^1.^ In model II adjusted for all except age.

**Table 5:** Crude and adjusted hazard ratio (HR) with 95% confidence interval (CI) for all-cause mortality within five years following a first percutaneous coronary intervention (PCI), among women and men, with disability pension (DP) or no DP (reference group), at time of revascularisation, for all and within each subpopulation.

	Women N = 10,804			Men N = 37,147		
	Crude	Model I	Model II	Crude	Model I	Model II
	HR (95% CI)	HR (95% CI)	HR (95% CI)	HR (95% CI)	HR (95% CI)	HR (95% CI)
**All**	3.07 (2.47–3.82)	2.83 (2.26–3.54)	2.25 (1.78–2.83)	2.16 (2.39–3.02)	2.34 (2.07–2.63)	1.95 (1.72–2.21)
**Age at intervention (years)** ^**1**^						
30–49	5.18 (2.97–9.01)		4.10 (2.25–7.48)	3.44 (2.36–5.02)		2.78 (1.89–4.09)
50–54	5.81 (2.98–11.30)		3.62 (1.75–7.45)	2.18 (1.57–3.02)		1.73 (1.23–2.43)
55–59	2.08 (1.37–3.14)		1.66 (1.08–2.56)	2.43 (1.99–2.98)		2.05 (1.66–2.54)
60–64	2.27 (1.63–3.16)		1.84 (1.31–2.58)	2.14 (1.79–2.57)		1.81 (1.49–2.19)
**Level of education**						
Elementary school	2.72 (1.98–3.73)	2.59 (1.87–3.58)	2.16 (1.55–3.01)	2.52 (2.13–2.98)	2.16 (1.81–2.57)	1.91 (1.60–2.29)
High school	3.72 (2.60–5.33)	3.35 (2.31–4.84)	2.53 (1.73–3.71)	2.70 (2.25–3.25)	2.37 (1.96–2.87)	2.03 (1.67–2.47)
College or university	2.58 (1.40–4.75)	2.32 (1.24–4.34)	1.44 (0.74–2.83)	2.79 (1.92–4.06)	2.51 (1.71–3.68)	1.87 (1.26–2.77)
**Country of birth**						
Sweden	3.14 (2.40–4.12)	3.00 (2.27–3.95)	2.52 (1.88–3.36)	2.93 (2.55–3.36)	2.58 (2.24–2.98)	2.30 (1.99–2.67)
Other	2.22 (1.53–3.22)	1.92 (1.31–2.83)	1.77 (1.20–2.62)	1.82 (1.47–2.25)	1.42 (1.14–1.77)	1.36 (1.09–1.71)
**Type of living area** (cities and communities)						
Large	3.08 (2.10–4.53)	2.77 (1.86–4.11)	2.23 (1.46–3.40)	2.56 (2.07–2.16)	2.18 (1.76–2.72)	1.95 (1.55–2.45)
Medium	3.33 (2.33–4.77)	3.08 (2.14–4.46)	2.38 (1.63–3.47)	2.66 (2.21–3.19)	2.34 (1.94–2.82)	1.87 (1.53–2.28)
Small	2.81 (1.89–4.16)	2.59(1.73–3.89)	2.09 (1.37–3.17)	2.88 (2.32–3.56)	2.53 (2.02–3.17)	2.13 (1.69–2.69)
**Year of intervention**						
1994–1997	3.89 (2.39–6.32)	3.67 (2.21–6.09)	3.10 (1.84–5.23)	2.55 (2.02–3.21)	1.17 (1.71–2.77)	1.88 (1.47–2.41)
1998–2001	2.19 (1.56–3.07)	1.91 (1.35–2.70)	1.54 (1.08–2.21)	2.52 (2.07–3.05)	2.32 (1.90–2.83)	1.88 (1.53–2.32)
2002–2006	3.72 (2.59–5.35)	3.78 (2.60–5.49)	2.72 (1.85–4.00)	3.20 (2.66–3.86)	2.84 (2.35–3.45)	2.03 (1.66–2.48)
**Indication for intervention**						
Acute Coronary Syndrome	2.53 (1.92–3.34)	2.30 (1.73–3.05)	1.79 (1.33–2.40)	2.60 (2.23–3.04)	2.26 (1.92–2.65)	1.84 (1.55–2.18)
Stable angina pectoris	3.90 (2.68–5.67)	3.55 (2.41–5.24)	2.94 (1.98–4.38)	2.63 (2.18–3.17)	2.30 (1.90–2.80)	1.97 (1.61–2.41)
Other/missing	7.19 (2.02–25.63)	7.60 (2.11–27.41)	12.45 (2.33–66.55)	4.48 (2.82–7.14)	3.87 (2.40–6.24)	3.54 (2.11–5.94)
**Diabetes Mellitus**						
Yes	2.68 (1.81–3.96)	2.49 (1.67–3.72)	2.37 (1.58–3.56)	2.76 (2.22–3.42)	2.53 (2.02–3.16)	2.30 (1.83–2.90)
No	2.87 (2.14–3.84)	2.58 (1.91–3.49)	2.19 (1.60–2.98)	2.52 (2.15–2.95)	2.16 (1.84–2.55)	1.90 (1.61–2.25)
Missing data	2.21 (1.11–4.37)	1.90 (0.92–3.90)	1.63 (0.75–3.51)	1.92 (1.41–2.63)	1.67 (1.21–2.30)	1.00 (0.68–1.47)
**In-patient care** (in the five years before intervention)						
Yes	3.66 (2.84–4.73)	3.40 (2.62–4.43)	2.68 (2.05–3.51)	2.77 (2.43–3.14)	2.42 (2.13–2.77)	2.09 (1.82–2.39)
No	1.62 (0.99–2.67)	1.47 (0.88–2.44)	1.15 (0.68–1.94)	1.87 (1.35–2.59)	1.59 (1.14–2.22)	1.30 (0.92–1.83)
**Re-intervention** (at least one within five years)						
Yes	3.07 (1.95–4.85)	3.06 (1.91–4.89)	2.56 (1.57–4.17)	2.75 (2.19–3.44)	2.50 (1.98–3.17)	2.29 (1.80–2.93)
No	3.10 (2.41–3.97)	2.78 (2.15–3.59)	2.15 (1.65–2.80)	2.69 (2.35–3.08)	2.32 (2.01–2.67)	1.84 (1.59–2.13)

Model I = Adjusted for Age; model II = Adjusted for: age, level of education, country of birth, type of living area, year of intervention, indication for intervention, diabetes mellitus, in-patient care within 5 years up to one day before intervention, and re-intervention.

^1.^ In model II adjusted for all except age.

Also in most of the studied subpopulations, regardless of type of intervention, gender and multivariable adjustments, those on DP at intervention had a higher HR for mortality. Patients on DP at the time of PCI in the lowest age-group (30–49 years) had a markedly high HR for mortality; especially women (HR 4.10; 95% CI 2.25–7.48).

Women on DP, with other or missing indication-data at time of PCI (n = 128) was another subpopulation with a markedly high HR for mortality (HR 12.45; 95% CI 2.33–66.55).

No associations were found between DP at time of intervention and mortality among patients (except men with CABG) with no in-patient care in the five years before intervention, and women with at least one re-intervention following CABG.

## Discussion

The aim of the present study was to investigate the five-year mortality following a first coronary revascularisation among women and men on DP compared with those not on DP at time of intervention, accounting for socio-demographic and medical factors. Of all patients who had survived the first month after the intervention 4% died within the five year following intervention (or through 31 December 2006). Regardless of type of intervention, gender and multivariable adjustments, the mortality was more than twice as high among patients on DP compared with no DP; also in most of the subpopulations.

To the best of our knowledge, no previous studies have reported mortality among patients on DP and not on DP at time of a first coronary revascularisation. However, previous studies of the general population have found mixed results regarding mortality after coronary revascularisation. One study found a two-year mortality of about 5% following coronary revascularisation performed during 1995–2004 [14]. However, the study had a shorter follow-up, and included both younger (<45 years) and older (85+) patients than included in the present study (30–64 years). Another study from Japan found a five-year mortality rate of 13% in women and 12% in men, following coronary revascularisation performed during 2000–2002 [15]. The report, contrary to the present study, included patients deceased within the 30 days after the intervention, and those older than 75 years of age.

CVD was the most common cause of death; which for the general population in 1997–2006 was the second largest [27]. This finding was not a surprise since all patients had had a history of coronary heart disease. The second largest cause of death was neoplasms; which for the general population was the number one cause of death in this age group. The third largest cause of death (for most patients) was metabolic, mainly diabetes mellitus. However, the third largest cause of death among patients with no DP at the time of PCI were external causes; in line with in the general population of those ages. A possible explanation for this finding could be the less severe disease and a less invasive treatment of these patients compared with the other studied patients, which makes them more alike the general population.

We found that patients on DP had a higher mortality compared with those not on DP at the time of the first coronary revascularisation, regardless of type of intervention, gender and after adjustments for socio-demographic and medical factors. This is in agreement with previous study results of the general population [19–25], adding that DP is an important indicator for premature death.

Also, in most of the described socio-demographic and medical subpopulations, patients on DP had a higher mortality than the reference group. Young patients (30–49 years) on DP at the time of PCI had a markedly high mortality compared to subjects in the same age-group without DP. This association could not be explained by socio-demographic and medical factors and is in accordance with previous findings of higher mortality in young individuals with DP, compared to those not on DP in the general population [25].

Women on DP and with other or missing indication-data for PCI had an extensively higher mortality compared with those not on DP. However, the 95% CI were wide since the size of this subpopulation was small. Nevertheless, the higher mortality could be due to a more advanced disease among these patients.

We did not find any association between DP and mortality among patients with no in-patient care in the five years before intervention except for in men with CABG. Regardless of DP, those without in-patient care could have had less co-morbidity and a less severe disease than those hospitalised before intervention. Future research on medical factors and the association between diagnose-specific DP and cause of death could clarify this further.

Moreover, no association between DP and mortality was found among women with at least one re-intervention following CABG. Since CABG is a more invasive treatment than PCI, and patients; especially women, often have a more severe coronary disease at the time of their first CABG, it might have been advanced disease and comorbidities, regardless of DP that had the main effect on the mortality risk in these patients.

### Strengths and limitations

As far as we know, this is the first nationwide, population-based cohort study exploring the five-year mortality among women and men in working age, on DP, or not on DP at the time of first coronary revascularisation. The main strengths of our study were the very large study population (n = 70,040), that all patients, aged 30–64 years with coronary revascularisation in Sweden between year 1994–2006 were included, the long follow-up period, that there were no losses to follow up, and the high quality of data from several different registers, including information on medical and socio-demographic factors [28]. Limitations may be the shorter follow up in those patients who had their revascularisation in the years 2002–2006, as mortality data only was available through 31 December 2006. Further, when stratifying for many factors in the Cox analyses, the size of some of the subpopulations (mostly regarding women) became small and generated wide confidence intervals. This could be due to the overall low representation of women in this study. Women are older at the onset of cardiovascular disease, and hence, they might either have been excluded from this working-age study, or they might have died within the 30 days following intervention, thus before the follow up in this study [14–17].

Another limitation may be the high percentage (12%) of missing data on diabetes mellitus. However, since many patients with heart disease also have co-morbidity we found it important to include all the available medical data on these patients. When we stratified for diabetes mellitus we specified the missing data in a third group.

## Conclusions

Regardless of type of intervention, gender and adjustments for socio-demographic and medical factors, patients on DP at time of intervention, had a higher mortality compared with those not on DP at the time of intervention.

## References

[1] Alexanderson K, Norlund A (2004) The Swedish Council on Technology Assessment in Health Care (SBU). Chapter 1. Aim, background, key concepts, regulations, and current statistics. Scand J Public Health Suppl 63:12–30. 15513650 10.1080/14034950410021808

[2] Henderson M, Glozier N, Holland Elliot K (2005) Long term sickness absence. BMJ 330: 802–803. 15817531 10.1136/bmj.330.7495.802 PMC556060

[3] OECD (2009) Sickness, Disability and Work, Breaking the barriers, Sweden: Will the recent reforms make it?: Directorate for Employment, Labour and Social Affairs, Organisation for Economic Co-operation and Development.

[4] (2013) Social Insurance in Figures 2012. Stockholm: Swedish Social Insurance Agency.

[5] Hamalainen H, Maki J, Virta L, Keskimaki I, Mahonen M, Moltchanov V, et al (2004) Return to work after first myocardial infarction in 1991–1996 in Finland. Eur J Public Health 14: 350–353. 15542868 10.1093/eurpub/14.4.350

[6] Perk J, Alexanderson K (2004) Swedish Council on Technology Assessment in Health Care (SBU). Chapter 8. Sick leave due to coronary artery disease or stroke. Scand J Public Health Suppl 63: 181–206. 15513657 10.1080/14034950410021880

[7] Bravata DM, Gienger AL, McDonald KM, Sundaram V, Perez MV, Varghese R, et al (2007) Systematic review: the comparative effectiveness of percutaneous coronary interventions and coronary artery bypass graft surgery. Ann Intern Med 147: 703–716. 17938385 10.7326/0003-4819-147-10-200711200-00185

[8] Hammar N, Sandberg E, Larsen FF, Ivert T (1997) Comparison of early and late mortality in men and women after isolated coronary artery bypass graft surgery in Stockholm, Sweden, 1980 to 1989. J Am Coll Cardiol 29: 659–664. 9060908 10.1016/s0735-1097(96)00531-1

[9] Norheim A, Segadal L (2011) Relative survival after CABG surgery is poorer in women and in patients younger than 70 years at surgery. Scand Cardiovasc J 45: 247–251. 10.3109/14017431.2011.582139 21604963

[10] Pilote L, Dasgupta K, Guru V, Humphries KH, McGrath J, Norris C, et al (2007) A comprehensive view of sex-specific issues related to cardiovascular disease. CMAJ 176: 1–44. 10.1503/cmaj.051455 PMC1817670 17353516

[11] Wijns W, Kolh P, Danchin N, Di Mario C, Falk V, Folliguet T, et al (2010) Guidelines on myocardial revascularization. Eur Heart J 31: 2501–2555. 10.1093/eurheartj/ehq277 20802248

[12] Yusuf S, Zucker D, Peduzzi P, Fisher LD, Takaro T, Kennedy JW, et al (1994) Effect of coronary artery bypass graft surgery on survival: overview of 10-year results from randomised trials by the Coronary Artery Bypass Graft Surgery Trialists Collaboration. Lancet 344: 563–570. 7914958 10.1016/s0140-6736(94)91963-1

[13] (2009) Årsrapport SWEDHEART 2008. RIKS-HIA, SEPHIA, SCAAR och Svenska Hjärtkirurgiregistret Uppsala (Yearly report, SWEDHEART 2008)(In Swedish). Uppsala Clinical Research Center.

[14] Blackledge HM, Squire IB (2009) Improving long-term outcomes following coronary artery bypass graft or percutaneous coronary revascularisation: results from a large, population-based cohort with first intervention 1995–2004. Heart 95: 304–311. 10.1136/hrt.2007.127928 19001000

[15] Funakoshi S, Furukawa Y, Ehara N, Morimoto T, Kaji S, Yamamuro A, et al (2011) Clinical characteristics and outcomes of Japanese women undergoing coronary revascularization therapy. Circ J 75: 1358–1367. 21483161 10.1253/circj.cj-10-0718

[16] Ortolani P, Solinas E, Guastaroba P, Marino M, Casella G, Manari A, et al (2013) Relevance of gender in patients with acute myocardial infarction undergoing coronary interventions. J Cardiovasc Med (Hagerstown) 14: 421–429. 22914306 10.2459/JCM.0b013e328357bb04

[17] Sato H, Kasai T, Miyauchi K, Kubota N, Kajimoto K, Miyazaki T, et al (2011) Long-term outcomes of women with coronary artery disease following complete coronary revascularization. J Cardiol 58: 158–164. 10.1016/j.jjcc.2011.03.003 21763108

[18] Zetterstrom K, Voss M, Alexanderson K, Ivert T, Pehrsson K, Hammar N, et al (2015) Prevalence of all-cause and diagnosis-specific disability pension at the time of first coronary revascularisation: a population-based Swedish cross-sectional study. PLoS One 10: e0115540 10.1371/journal.pone.0115540 25629517 PMC4309573

[19] Bjorkenstam C, Alexanderson K, Bjorkenstam E, Lindholm C, Mittendorfer-Rutz E (2014) Diagnosis-specific disability pension and risk of all-cause and cause-specific mortality—a cohort study of 4.9 million inhabitants in Sweden. BMC Public Health 14: 1247 10.1186/1471-2458-14-1247 25476556 PMC4289270

[20] Jonsson U, Alexanderson K, Kjeldgard L, Westerlund H, Mittendorfer-Rutz E (2013) Diagnosis-specific disability pension predicts suicidal behaviour and mortality in young adults: a nationwide prospective cohort study. BMJ Open 3. 10.1136/bmjopen-2012-002286 PMC3586126 23396561

[21] Gjesdal S, Haug K, Ringdal P, Maeland JG, Hagberg J, Roraas T, et al (2009) Sickness absence with musculoskeletal or mental diagnoses, transition into disability pension and all-cause mortality: A 9-year prospective cohort study. Scand J Public Health 37: 387–394. 10.1177/1403494809103994 19324926

[22] Gjesdal S, Svedberg P, Hagberg J, Alexanderson K (2009) Mortality among disability pensioners in Norway and Sweden 1990–96: Comparative prospective cohort study. Scand J Public Health 37: 168–175. 10.1177/1403494808100937 19179451

[23] Gjesdal S, Maeland JG, Svedberg P, Hagberg J, Alexanderson K (2008) Role of diagnoses and socioeconomic status in mortality among disability pensioners in Norway—a population-based cohort study. Scand J Work Environ Health 34: 479–482. 19137210 10.5271/sjweh.1286

[24] Karlsson NE, Carstensen JM, Gjesdal S, Alexanderson KA (2007) Mortality in relation to disability pension: findings from a 12-year prospective population-based cohort study in Sweden. Scand J Public Health 35: 341–347. 17786796 10.1080/14034940601159229

[25] Wallman T, Wedel H, Johansson S, Rosengren A, Eriksson H, Welin L, et al (2006) The prognosis for individuals on disability retirement. An 18-year mortality follow-up study of 6887 men and women sampled from the general population. BMC Public Health 6: 103 16630360 10.1186/1471-2458-6-103 PMC1459134

[26] Jernberg T, Attebring MF, Hambraeus K, Ivert T, James S, Jepsson A, et al (2010) The Swedish Web-system for Enhancement and Development of Evidence-based care in Heart disease Evaluated According to Recommended Therapies (SWEDEHEART). Heart 96: 1617–1621. 10.1136/hrt.2010.198804 20801780

[27] (2015) Dödsorsaker (cause of death). Socialstyrelsen (The National Board of Health and Welfare).

[28] Ludvigsson JF, Andersson E, Ekbom A, Feychting M, Kim JL, Reuterwall C, et al (2011) External review and validation of the Swedish national inpatient register. BMC Public Health 11: 450 10.1186/1471-2458-11-450 21658213 PMC3142234

